# Macro‐ and Microanatomy of the Sympathetic Innervation of the Spleen in Rodents

**DOI:** 10.1002/cne.70086

**Published:** 2025-08-29

**Authors:** Maria Moura, Alice Miranda, Jonas Campos, Andreia G. Pinho, Sara Rito‐Fernandes, Carina Soares‐Cunha, António J. Salgado, Nuno A. Silva, Susana Monteiro

**Affiliations:** ^1^ Life and Health Sciences Research Institute (ICVS), School of Medicine University of Minho Braga Portugal; ^2^ ICVS/3B's Associate Lab PT Government Associated Lab Braga/Guimarães Portugal

**Keywords:** celiac ganglion, macroscopy, microstructure, spleen, sympathetic neuron

## Abstract

In recent years, several studies have demonstrated the crucial role played by the sympathetic spleen innervation in regulating immune cell function involving fighting pathogens or tissue injury. These findings have sparked interest across different research fields with a common goal of understanding and manipulating splenic sympathetic activity to modulate immune function and inflammation. However, the anatomical identification of spleen‐projecting neurons in rodents presents a considerable challenge, given the multi‐compartmentalized location of their cellular components.

This article addresses this challenge by providing a detailed anatomical dissection guide of the mouse celiac ganglion and splenic nerve, harboring the cell body and projecting axons, respectively. By combining antero‐ and retrograde neuronal tracing, immunofluorescence, 3D reconstruction, and viral tracing techniques, we validate the connectivity between the celiac ganglion and the spleen and provide insights into their microanatomy. Importantly, we demonstrate the feasibility of viral transduction in these neurons. Additionally, we identified nerve‐associated macrophages (NAMs) within the splenic nerve and demonstrated their responsiveness to inflammatory stimuli. Our findings offer a comprehensive anatomical framework for studying spleen‐projecting neurons, paving the way for future investigations into their role in immune regulation and inflammation, as well as their manipulation using advanced neurobiological tools.

## Graphical abstract

The multi‐compartmental organization of spleen‐projecting neurons—from the celiac ganglia to the spleen—complicates anatomical identification. We provide a dissection guide, characterize their microstructure, and use neuronal tracing to validate the celiac ganglia–spleen connection while demonstrating that these neurons can be transduced by a viral vector.

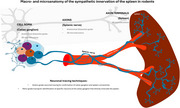

## 1 Introduction

Sympathetic nerve supply to lymphoid organs enables localized secretion of catecholamines and neuropeptides, which are crucial for controlling immune responses (reviewed in Bellinger et al. 2008). These neuro‐immune communications are vital for maintaining immune homeostasis and orchestrating responses to injury or infection. The spleen is densely populated by sympathetic noradrenergic fibers and terminals throughout its parenchyma, with the nerve fibers displaying a panicle‐like morphology. In contrast, sympathetic fibers in the thymus or lymph nodes are typically restricted to a more superficial distribution (Ding et al. 2019). This enriched neuronal innervation of the spleen, compared to other lymphoid organs, suggests that the spleen is uniquely positioned as a hub for neuroimmune interactions. The spleen receives its sympathetic innervation from the splenic nerve plexus surrounding the branches of the splenic artery. When entering the spleen, these fibers are found within the adventitia of central arteries in the white pulp and associated with arterioles in the red pulp (Verlinden et al. 2019). Cell bodies of spleen‐innervating neurons are located outside the spleen in the celiac ganglia (Quinson et al. 2001), one of the largest prevertebral ganglia. These ganglia act as a relay, transmitting signals from central preganglionic neurons to peripheral postganglionic neurons, which then innervate the spleen. Preganglionic inputs can be modified at the ganglia, therefore modulating the neuronal excitability of postganglionic neurons (Miolan and Niel 1996).

The activity of the sympathetic innervation of the spleen has attracted significant attention in the last decade due to its crucial role in host defense from pathogens and wound healing (Ding et al. 2019; Carnevale et al. 2016), as well as its involvement in conveying the cholinergic anti‐inflammatory reflex (Rosas‐Ballina et al. 2011). However, conflicting findings on the role of sympathetic activity in immune responses underscore the need for further investigation. For instance, while sympathetic activity has been historically associated with anti‐inflammatory responses or immunosuppression (Ueno et al. 2016; Wong et al. 2011; Y. Zhang et al. 2013), recent studies have shown that decreasing peripheral sympathetic activity during viral infections can lower proinflammatory cytokines and improve survival rates (Grebe et al. 2010). Yet, chemical sympathectomy, although effective in reducing acute inflammation, surprisingly intensified inflammation at later stages (Schaible and Straub 2014), revealing a more complex role of the sympathetic regulation of immune responses than previously understood. These inconsistencies highlight a complex context‐ and time‐dependent role for sympathetic innervation in immune regulation, underscoring the need for further studies that manipulate the spleen innervation activity.

The functional role of the sympathetic‐spleen connection is mediated largely through norepinephrine (NE) release from sympathetic terminals, acting on adrenergic receptors expressed by immune cells. NE modulates immune responses in a context‐ and concentration‐dependent manner, influencing cytokine production, immune cell trafficking, and inflammation resolution (Bellinger et al. 2008). However, the precise mechanisms underlying sympathetic‐immune interactions remain unclear, particularly at the level of specific neurons that project to the spleen. A better anatomical and functional characterization of these neurons is crucial for targeted neuromodulation approaches.

Techniques such as splenic nerve ablation or neuromodulation of its activity, using methods like chemogenetics or optogenetics, may be crucial for fully understanding how spleen innervation shapes immune responses. However, when compared to neuromodulation research in the brain, the manipulation of peripheral neurons using advanced molecular and genetic technologies is still at an early stage. The complex multi‐compartmentalized location of the spleen‐projecting neurons, with their cell bodies in the celiac ganglia, projecting axons surrounding the splenic artery, and terminals in the spleen parenchyma, presents a challenge in its anatomical identification. This is particularly pronounced in the mouse, one of the most commonly used preclinical research models, owing to its small size.

This article presents an easy step‐by‐step guide for facilitating the macroscopic identification of the celiac ganglia and splenic nerve in mice. Additionally, we provide a detailed description of their microstructure using immunofluorescence and antero‐ and retrograde tracing techniques and demonstrate the feasibility of transducing these neurons using a viral vector.

## 2 Material and Methods

### 2.1 Animals

The Ethical Subcommittee in Life and Health Science (SECVS; 018/2019, University of Minho), ORBEA (EM/ICVS‐I3Bs_017‐2021), and Direcção Geral de Veterinária (DGAV) (DGAV:106397/24‐S) previously approved all experiments. Local regulations on animal care and experimentation (European Union Directive 2010/63/EU) were respected. 10–15‐week‐old male Wistar (Charles River; RRID:RGD_2308816) rats, C57BL6/J (Charles River; RRID:IMSR_CRL:027) and heterozygous CX3CR1^eGFP^ (Charles River; RRID:IMSR_JAX:005582) mice were used. Animals were housed in an animal facility, maintained under an artificial 12 h light/dark cycle with lights on from 8:00 a.m. to 8:00 p.m., a room temperature of 21 ± 1°C, and 50%–60% relative humidity. Ad libitum standard diet (Mucedola, Italy) and water were provided. Animals were housed in filter‐topped polysulfone cages in groups of six animals (mice) or two animals (rats) in cages with 370 or 820 cm^2^ of floor area, respectively (Techniplast, USA). Corncob bedding, soft tissue paper, and cardboard tubes were used for environmental enrichment. The number of animals in each experiment is indicated in the respective experiment protocol.

### 2.2 Anatomical Dissections

Rodents were deeply anesthetized before anesthesia overdose by an intraperitoneal administration of ketamine (Ketamidor, VetViva Richter GmbH; 75 mg/kg) and medetomidine (Sededorm, Vetpharma Animal Health, S.L.; 1 mg/kg). Reflexes were evaluated by toe pinching, and after death confirmation by a physical method, spleen, splenic artery, and ganglia dissections were conducted. All anatomical dissections were performed under a stereomicroscope (A60F, Leica Microsystems, Germany). Briefly, a midline abdominal incision was made to expose the abdominal cavity. Visceral organs, including the spleen, were retracted laterally to expose the left kidney. The celiac ganglia were located between the celiac artery and the superior mesenteric artery, at the level of their origin from the abdominal aorta. The splenic artery was dissected proximal to its origin from the celiac trunk until the very first bifurcation towards the spleen. The spleen was dissected afterward to preserve anatomical landmarks needed for the celiac ganglia and splenic artery dissections. The stellate ganglia, which are located dorsolateral to the longus colli muscle that lies just ventral to the transverse processes of the seventh cervical and first thoracic vertebrae, were also removed as a nonrelevant ganglion for the spinal‐to‐spleen circuit. For this protocol, we used two mice and two rats.

### 2.3 Cryostat Sectioning

The spleen and ganglia were collected and postfixed by placing them in 4% (w/v) paraformaldehyde (PFA) for 24 h, at 4°C in the dark. On the following day, the organs were cryopreserved in 30% (w/v) sucrose solution at 4°C until they reached saturation. The organs were then immersed in a plastic mold filled with optimal cutting temperature compound (OCT, Sakura Finetek, USA), placed in isopentane‐soaked cotton, snap‐frozen using liquid nitrogen, and stored at −20°C until downstream assays. The spleen and ganglia were sectioned on a cryostat (CM1900, Leica Biosystems; RRID:SCR_020218), at −20°C, into 20 µm sequential sections and thaw‐mounted on microscopic slides. The spleen was cut in a long‐axis parasagittal manner. Slides were left to dry for 1 h at RT, in the dark, before storing at −20°C until use.

### 2.4 Immunofluorescence

Spleen sections were immunostained for tyrosine hydroxylase (TH) to identify catecholaminergic nerve fibers and terminals. Ganglia sections were double‐stained with TH (Millipore Cat# AB152, RRID:AB_390204) (for catecholaminergic neuronal cell bodies) and norepinephrine transporter (NET) (Thermo Fisher Scientific Cat# MA5‐24647, RRID:AB_2637262) for (noradrenergic neuronal cell bodies and fibers). Thaw‐mounted sections were left in PBS 1× for 30 min to remove OCT residues. Subsequently, sections were permeabilized in PBS 1× supplemented with 0.5% Triton X‐100 (PBS‐T) for 30 min, followed by 30 min blocking, after rinsing in PBS 1× three times, with 5% (v/v) Fetal Calf Serum (FCS, Abcam, UK) in 0.5% PBS‐T, at RT. Sections were then incubated O/N, at 4°C, with the primary antibodies prepared in 5% FCS in 0.5% PBS‐T (detailed information on antibodies can be found in Table 1). On the following day, sections were rinsed in PBS 1× and incubated for 2 h with the respective secondary antibodies (see Table 1) prepared in 5% FCS in 0.5% PBS‐T at RT. After three rinses with PBS 1×, sections were mounted with PermaFluor (Thermo Scientific) and then stored at 4°C, protected from the light. Negative controls were used to ensure the specificity of the staining. For this experiment, an *n* = 4 was used for each species (mouse and rat).

**TABLE 1:** Detailed list of primary and secondary antibodies used in the immunofluorescence staining.

Marker	Target	Dilution	Reference	Supplier	RRID
Primary antibodies					
Norepinephrine transporter monoclonal antibody	Norepinephrine transporter	1/200	MA5‐24647	Invitrogen, USA	AB_2637262
Anti‐tyrosine hydroxylase	Sympathetic fibers	1/1000	AB152	Milllipore, USA	AB_390204
CD68	Lysosomal membrane of myeloid cells	1/500	14068182	Invitrogen	AB_2572857
Secondary antibodies					
Alexa Fluor 488 goat anti‐mouse		1/1000	A11029	Invitrogen, USA	AB_2534088
AlexaFluor 594 goat anti‐rabbit		1/1000	A11037	Invitrogen, USA	AB_2534095
AlexaFluor 647 goat anti‐rat		1/1000	A21247	Invitrogen, USA	AB_141778

### 2.5 Whole‐Mount Immunofluorescence for Three‐Dimensional Imaging of the Splenic Artery and Celiac Ganglion

After careful dissection, the splenic artery was placed in 4% PFA for 1 h, in the dark and washed with Phosphate Buffered Saline (PBS) 1X. After fixation, arteries were individually placed in 96‐well plates and followed the same IF protocol as described above for tissue sections. Briefly, the arteries were permeabilized in PBS 1× supplemented with 0.5% Triton X‐100 (PBS‐T) for 30 min, followed by 30 min blocking, after rinsing in PBS 1× three times, with 5% (v/v) FCS (Abcam, UK) in 0.5% PBS‐T, at RT. The arteries were then incubated O/N, at 4°C, with the primary antibodies prepared in 5% FCS in 0.5% PBS‐T (detailed information on antibodies can be found in Table 1). On the following day, the arteries were rinsed in PBS 1× and incubated for 2 h with the respective secondary antibodies (see Table 1) prepared in 5% FCS in 0.5% PBS‐T at RT. After three rinses with PBS 1×, arteries were mounted with PermaFluor (Thermo Scientific) and then stored at 4°C, protected from the light. A celiac ganglion was dissected from the same mouse for a whole‐mount immunofluorescence using the TH marker in order to visualize the neuronal bodies and axonal projections in a three‐dimensional (3D) manner, as described in McKinnon et al. (2019). Briefly, after isolation and fixation, the left celiac ganglion was washed in PBS 1× supplemented with 0.5% Triton X‐100 (PBS‐T) O/N at 4°C. The celiac ganglion was then incubated for 5 days, at 4°C, with the primary antibody prepared in 5% FCS in 0.5% PBS‐T (detailed information on antibodies can be found in Table 1). After the incubation period, the celiac ganglion was washed three times for a period of 2 h each in 0.5% PBS‐T and incubated for 3 days with the respective secondary antibody (see Table 1) prepared in 5% FCS in 0.5% PBS‐T, at 4°C. After a final wash in PBS‐T for 2 h and another two washes in 50 mM Tris‐HCl for 1 h each, the celiac ganglion was mounted with PermaFluor (Thermo Scientific) and then stored at 4°C, protected from the light.

### 2.6 Spleen Tissue Clearing and Immunofluorescence

For this protocol, one mouse was transcardially perfused first with 25 mL of PBS 1× with heparin (10 units per mL) at 4°C and then with 50 mL of 4% PFA at 4°C. The spleen was carefully dissected and washed with heparin/PBS solution at 4ªC for 30 min and then postfixed with 4% PFA for 24 h, at 4°C.

Before embedding the organ in a 2% agarose (w/v) solution in PBS, the spleen was once again washed three times in PBS 1× for 1 h each. The tissue was sectioned using a vibratome at 500 µm thickness.

The spleen sections were permeabilized and blocked with 500 µL of Ce3D Permeabilization/Blocking Buffer (Biolegend, USA) at RT with gentle shaking for 2 days. This step is crucial for the permeabilization of the cell membrane and for preventing the nonspecific binding of antibodies. The sections were stained with primary antibody TH prepared in 500 µL Ce3D Antibody Diluent Buffer (Biolegend, USA) for 2 days at RT. After incubation, sections were rinsed with Ce3D Wash Buffer (Biolegend, USA) three times for 8 h and incubated for 2 days at RT with the respective secondary antibody, followed by another rinse cycle with Ce3D Wash Buffer (Biolegend, USA).

The sections were left to clear for 24 h in 500 µL of Ce3D Tissue Clearing Solution (Biolegend, USA) at RT before mounting the splenic section on the microscopical slide using the tissue clearing solution as a mounting medium. Performing a clearing of the sample provides the ability to study the tissue in its 3D state by matching the refractive index (the sample will appear transparent).

### 2.7 Widefield and Confocal Imaging

Images of immunostained sections were acquired using an Olympus Widefield Upright Microscope BX61 (Olympus BX61 Upright Wide Field Microscope [RRID:SCR_020343]). Immunostained ganglia and spleen sections were all acquired, and the most representative image was selected.

The immunostained whole‐mount preparation of the splenic artery and celiac ganglia was acquired using a confocal point‐scanning microscope, Olympus FV1000 (Olympus FV1000 Confocal Microscope [RRID:SCR_020337]) with a 20× air objective with a numerical aperture NA 0.75. Mosaic Z‐stacked images were captured with 5 µm step size and used for 3D reconstruction using the FV10‐ASW 2.0c software.

For whole‐mount imaging of the cleared spleen, acquisitions were made using a 10× air objective with NA 1.0. Using an automatic stage, *x*,*y*,*z* mosaics were acquired with a 10‐µm step size for a total of 660 µm over the *z*‐axis. Images had a resolution of 0.8045 pixels per micron and a 3D voxel size of 1.2430 × 1.12430 × 103. The whole spleen was scanned to allow visualization of both arterial insertions as well as patterns of adrenergic innervation. For visualization, images were processed with 3D maximal projections.

### 2.8 Analysis of Splenic Nerve‐Associated Macrophages

To investigate the impact of an inflammatory stimulus on CX3CR1^+^ macrophages associated with the splenic Nerve‐associated Macrophages (NAMs), mice were intraperitoneally injected with LPS (250 µg/kg; *n* = 3). Saline‐injected mice (*n* = 3) were used as controls. After 1 h, mice were sacrificed. To examine NAMs in the splenic nerve, the splenic artery was dissected from its origin at the celiac trunk to its first bifurcation, yielding a Y‐shaped sample (Figure 5A, dashed box B). After fixation in 4% PFA for 1 h and immunofluorescence staining for TH and CD68 (as described above), arteries were stored in PBS at 4°C. Confocal z‐stacks were acquired using identical laser and acquisition settings. For each artery, four to six non‐overlapping fields were imaged per animal. Each data point in the graphs represents the mean of all microphotographs analyzed per animal. Image acquisition and analysis were performed blinded to experimental condition. Data from one saline‐injected animal were excluded due to displacement of the artery out of the imaging plane during confocal acquisition.

NAM density was quantified as the number of CX3CR1⁺ cells per mm^2^ of nerve area using ImageJ. For each image, the total nerve area was segmented based on TH staining to normalize NAM counts. NAMs were manually counted based on GFP signal co‐localized with DAPI within TH^+^ areas. NAM soma size was estimated by measuring the area of 5–10 randomly selected NAMs per field. To assess NAM activation, the CD68+ area within NAMs was quantified using threshold‐based segmentation. Group comparisons were performed using unpaired two‐tailed Student's *t*‐tests (GraphPad Prism). Data are presented as mean ± SEM.

### 2.9 Neuronal Tracing

Two different experimental designs were tested to trace specific spleen‐innervating postganglionic neurons. A retrograde approach, involving tracer injection at the spleen, and an anterograde approach, involving a viral tracer injecting in the left celiac ganglion, were used.

Two mice were anesthetized as described previously. An abdominal wall incision was performed to access the spleen or the left celiac ganglion, depending on the approach.

Hydroxystilbamidine bis (methanesulfonate) (Sigma Aldrich, Germany), also known as Fluoro‐Gold, was used for retrograde tracing. A solution of 4 mg/mL Fluorogold was prepared in sterile 0.9% saline and then filtered through a syringe filter. Using a Hamilton syringe equipped with a 30G needle, a “freehanded” injection of 2 µL of Fluorogold solution was given, 1 µL in two different locations of the spleen near the arterial insertions, to target splenic nerve branches and terminals. The syringe was only retracted 1 min postinjection, and the injection site was cleaned with a Q‐tip to prevent tracer contamination/leakage.

For anterograde tracing, a viral vector was used. Specifically, an adeno‐associated virus (AAV) that mediates the expression of tdTomato (codon diversified) under the CAG promoter (AAV1‐CAG‐TdTOMATO (Addgene, 59462‐AAV1, RRID:Addgene_59462). A solution of 2 µL of AAV1‐CAG‐TdTOMATO (titer ≥ 5 × 10^12^ vg/mL) was “freehandedly” injected into the left celiac ganglion of two animals. The syringe was only retracted 1 min postinjection, and the injection site was cleaned with a Q‐tip to prevent tracer contamination/leakage. Following injection, the abdominal wall and skin were closed using absorbable suture. The celiac ganglion and the spleen were harvested 7 days after retrograde tracer injection and 2 months after viral tracer injection for tracer confirmation. The stellate ganglion was harvested and used as a control for nonspecific tracing. The samples were processed for cryostat sectioning, as mentioned previously, and then section images were acquired using an Olympus Widefield Upright Microscope BX61 (Olympus, USA; RRID:SCR_020343).

## 3 Results

### 3.1 Anatomical Location of the Mouse Celiac Ganglia

**FIGURE 1:**
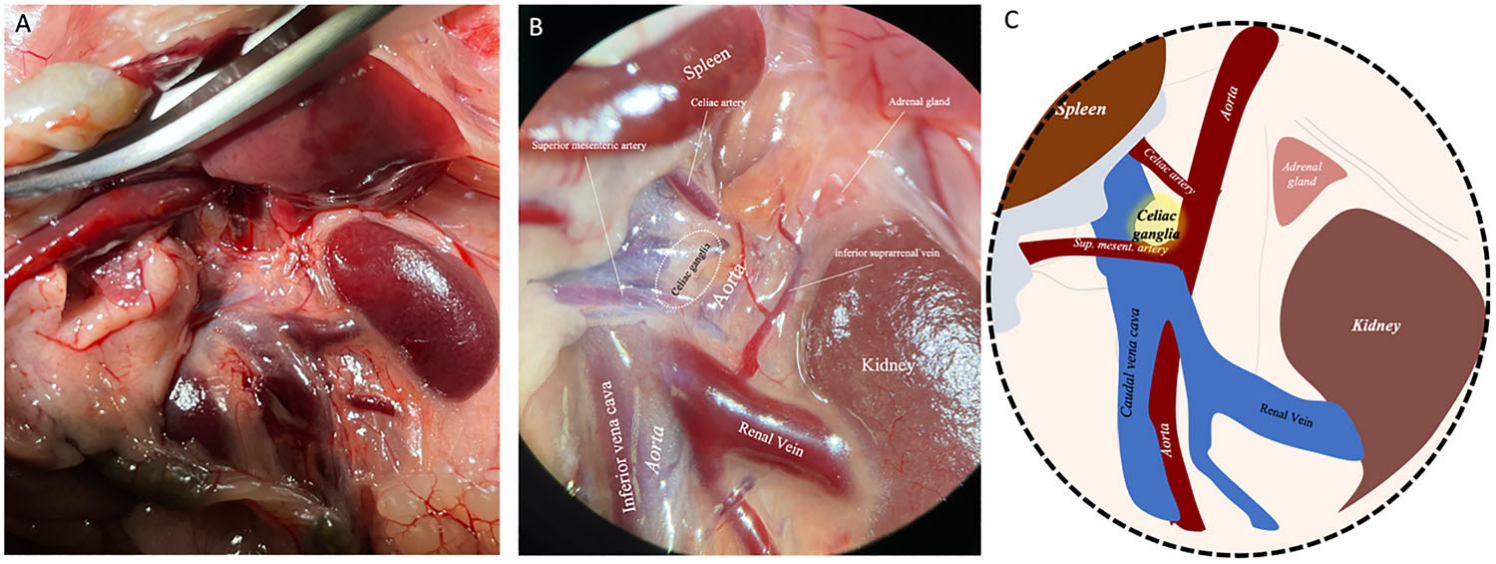
Anatomical dissection of the celiac ganglion in the mouse. (A–C) Celiac ganglion dissection procedure. Mice were euthanized, a midline incision was made, and (A) the viscera were retracted from the abdominal cavity. (B) Anatomical identification and localization of the celiac ganglion. The celiac ganglion is in the upper abdomen near the celiac artery branches from the abdominal aorta. (C) Schematic anatomic representation of the celiac ganglion localization in the mouse.

Cell bodies of spleen‐projecting neurons are located outside the spleen, in the celiac ganglia—a collection of neuronal cell bodies projecting to several organs. The celiac ganglia are composed of two adjacent ganglia (left and right) overlying the abdominal aorta, which, at this particular level, rests at a considerable depth within the abdominal cavity. A midline skin incision was performed to access the abdominal cavity, followed by an abdominal wall incision. In order to access the level of the celiac ganglia, the visceral organs, including the spleen, were laterally retracted towards the right side of the animal. This retraction continued until the left kidney became fully exposed (Figure 1A,B). Importantly, this retraction approach will primarily expose the left celiac ganglion, while the right celiac ganglion will be situated beneath it. The renal vein is a valuable anatomical landmark due to its considerable size and clear insertion into the kidney. This prominent anatomical feature can aid in identifying the abdominal aorta, which courses beneath the renal vein and runs parallel to the caudal vena cava (Figure 1B,C). At the level of the adrenal gland and the cranial pole of the kidney, the aorta gives off two large arterial branches, the celiac artery and the superior mesenteric artery (Figure 1B,C). The celiac ganglia are positioned in the periaortic space between these two arteries (Figure 1B,C). The celiac ganglion may not be macroscopically noticeable in the mouse due to its small size. As a result, the contiguous tissue between the two arteries containing the celiac ganglion was carefully collected with forceps and further processed for histological analysis to confirm the correct isolation of the ganglion. Given the anatomical similarities between rats and mice, initial training in rats facilitated the identification of small structures like the celiac ganglion, making the transition to mice more effective. After dissection, ganglia from both species were processed for immunohistochemistry and microscopy to analyze their microstructure.

### 3.2 Microstructural Characterization of the Spleen‐Projecting Neurons

The microstructure of the dissected tissue in the mouse and rat was analyzed by immunofluorescence using neuronal markers for sympathetic neurons to confirm its neuronal nature. The stellate ganglion, a relatively straightforward ganglion to identify due to its larger volume and distinctive elongated morphology, was used as a standard for the expected ganglion microstructure.

**FIGURE 2:**
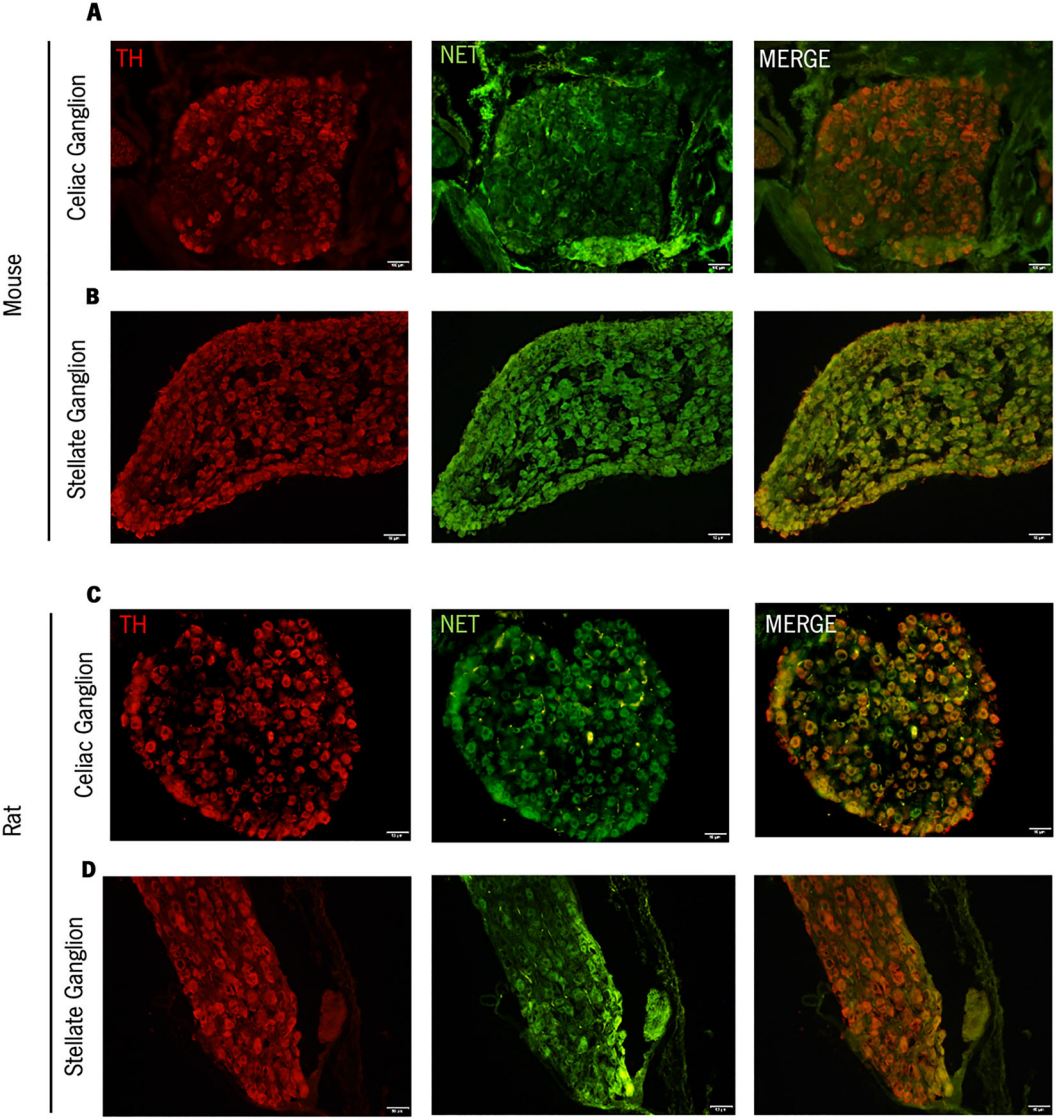
Microstructural characterization of the mouse and rat celiac and stellate ganglia. (A–B) Immunofluorescence characterization of the mouse celiac (A) and stellate (B) ganglia. Both ganglia are stained for NET (green) and TH (red). (C–D) Immunofluorescence characterization of rat celiac (C) and stellate (D) ganglia. Both ganglia are stained for NET and TH.

Confirming the macroscopic analysis, the overall microstructure of the celiac ganglion differs slightly from that of the stellate ganglion. The celiac ganglion (both in rat and mouse) presents a rounder shape (Figure 2A and C) when compared with the stellate ganglion (Figure 2B and D), which presents a larger and elongated, sharper shape.

Neurons from the celiac and stellate ganglia express both the sympathetic marker TH and the noradrenergic marker, NET (Figure 2). As expected, TH‐positive neurons overlap with NET‐positive neurons, hence confirming the noradrenergic nature. In line with the similar anatomy between mice and rats, no major differences were found in the cytoarchitecture of the celiac and stellate ganglia when comparing species. This structural similarity supports our approach of using rats for initial training in ganglia dissection before applying the procedure to mice.

**FIGURE 3:**
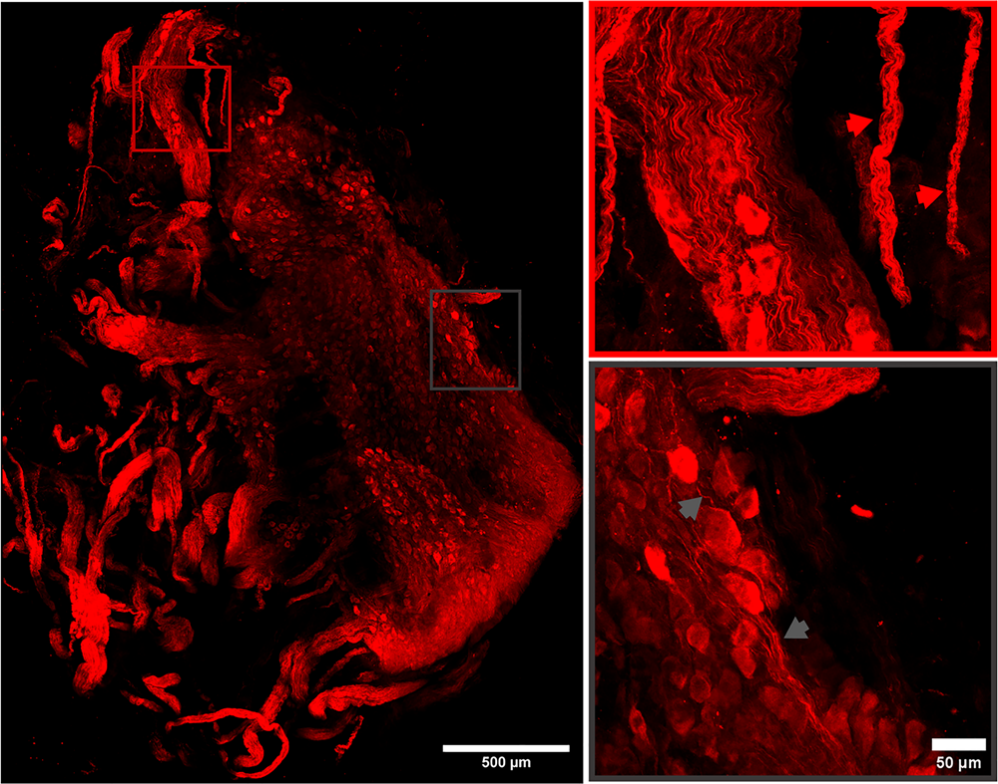
3D reconstruction of a whole‐mount immunostained mouse celiac ganglion. Confocal imaging of a whole‐mount of the mouse celiac ganglion stained for tyrosine hydroxylase (TH). Reconstruction of z‐stack images of the mouse celiac ganglia through confocal imaging immunostained for tyrosine hydroxylase (TH), in which we can visualize sympathetic cell bodies and departing axonal fibers (gray arrows) organized in nerve bundles (red arrows).

We then performed a whole‐mount immunostaining of the mouse celiac ganglion (Figure 3) for 3D visualization. This technique, in comparison to the conventional imaging of the sectioned celiac ganglion, preserves the natural 3D architecture, the spatial distribution of sympathetic neurons, and their anatomical connections, minimizing sectioning‐associated distortion or damage. The celiac ganglion is densely populated by the large soma of sympathetic neurons (Figure 3). It is also possible to observe the spatial distribution of sympathetic neuron projections as multiple processes emanating from the soma (Figure 3, gray insert and arrows), including fine axonal segments that are then organized in nerve bundles (Figure 3, red insert and arrows).

**FIGURE 4:**
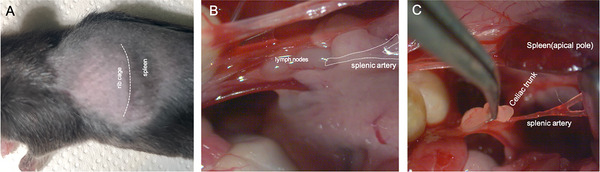
Anatomical dissection of the splenic artery in the mouse. (A) A left flank skin incision is made along the border between the rib cage and spleen (dashed white line). (B) Using a retractor, the spleen is separated from the liver and stomach (retracted upward) exposing at a lower plane a collection of lymph nodes. These lymph nodes can be used as an anatomical landmark since the splenic artery branching from the celiac trunk courses beneath the pancreas at this point (dashed white line drawing depicting the location of the splenic artery branching). (C) Dissected splenic artery branching from the celiac trunk and its relation to the lymph nodes.

The splenic nerve is a collection of axonal bundles leaving the celiac ganglion towards the spleen. The splenic nerve is organized in nerve fascicles surrounding the splenic artery. The splenic artery stems from the celiac artery, bifurcating several times until it reaches the spleen hilum. To identify the initial branch of the splenic artery, a skin incision was made in the left flank of the mouse, in alignment with the spleen position (Figure 4A). Taking advantage of the trichotomy, the dark red color of the spleen becomes visible beneath the skin, orienting the skin incision (Figure 4A). The abdominal wall was then carefully opened. Using a retractor, the spleen and pancreas were carefully moved away from the stomach and liver (Figure 4C). The splenic artery branching is hidden by the pancreas (Figure 4B). The initial branching point of the splenic artery from the celiac artery is located at a lower plane. A cluster of lymph nodes can serve as an anatomical landmark for identifying this branch point (Figure 4B). The initial portion of the splenic nerve, until its first bifurcation towards the spleen, was carefully separated from the pancreas (Figure 4C).

**FIGURE 5:**
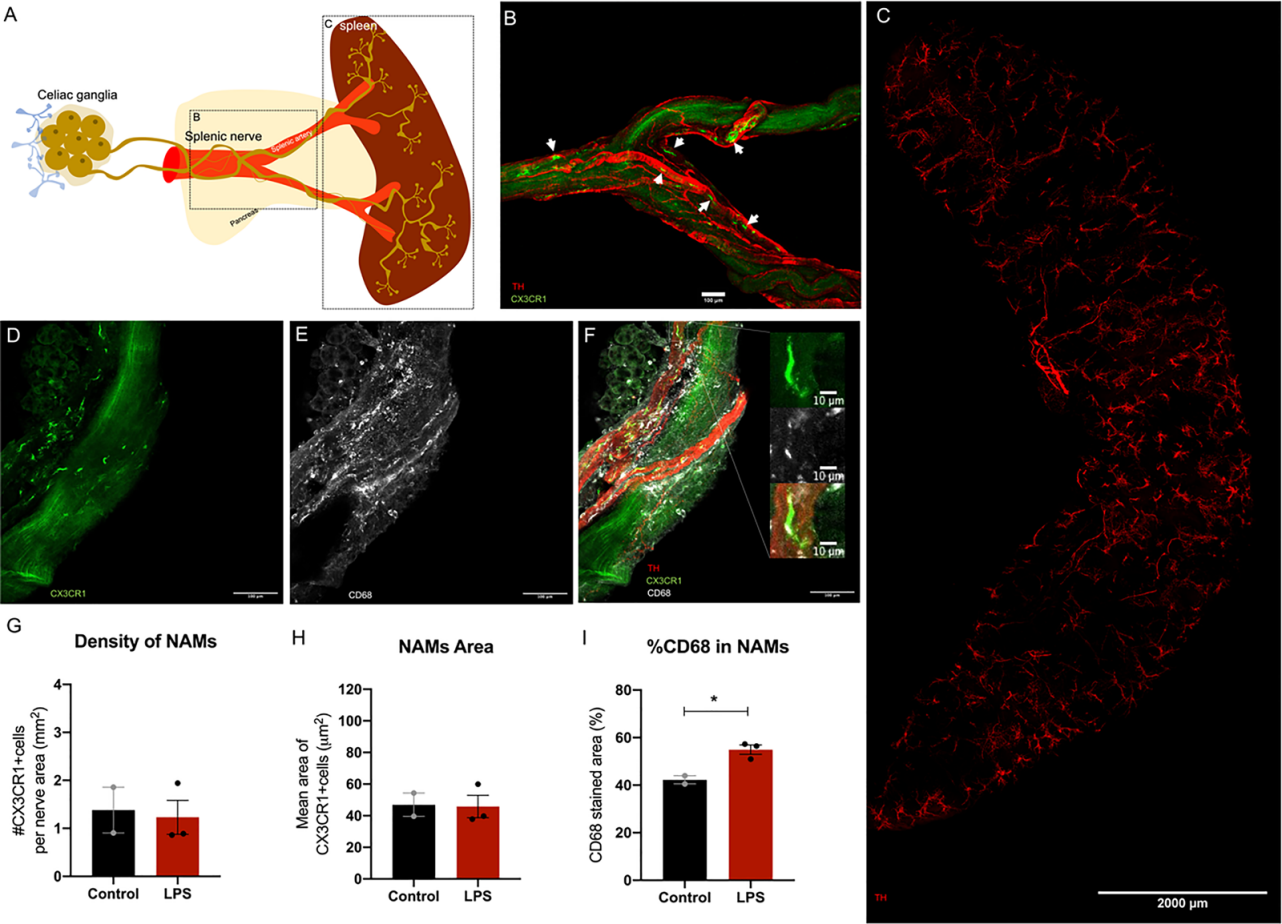
Microstructural characterization of the spleen‐projecting nerve fibers and terminals (A) Schematic representation of the spleen‐projecting neurons located in the celiac ganglion, with axons running along the several bifurcations of the splenic artery and terminals in the spleen. (B) Whole‐mount image of the splenic artery from a CX3CR1^GFP^ animal. The artery section was dissected from its origin at the celiac trunk to its first bifurcation. TH‐positive fibers (red) demonstrate the location of the splenic nerve around the artery, with GFP+ cells associated with nerve fibers (white arrows) suggestive of nerve‐associated macrophages (NAMs). (C) Cleared spleen displaying TH‐positive nerve bundles inserting at the spleen hilum and a dense fiber distribution across the spleen parenchyma. (D–F) Whole‐mount images of the splenic artery showing that NAMs express CD68. (D) NAMs express CX3CR1 (green) and display a unique elongated morphology and distribution on the surface of the artery. (E) Distribution of the lysosomal marker CD68 (white) along the splenic artery. (F) The merged image confirms CD68 expression (white) in CX3CR1^+^ NAMs (green), which are associated with TH‐positive nerve fibers (red). (G–I) Quantification of NAMs density normalized to TH‐positive area (G), total area (H), and % of CD68 stained area (I) in control versus LPS‐treated conditions. Data points represent mean values from averaged microphotographs per animal from a total of two to three animals per group. Data expressed as SEM *p < 0.05.

We performed a whole‐mount immunofluorescence on the splenic artery to visualize projecting TH‐positive axons from postganglionic neurons that innervate the spleen (Figure 5A–F). The splenic nerve consists of TH‐positive fine nerve fibers and large nerve bundles that surround and run along the splenic artery (Figure 5B and F). Along with TH^+^ fibers, we observed cells positive for CX3CR1 (Figure 5B,D and F) that were associated with nerve fibers and exhibited an elongated morphology. While CX3CR1 is also expressed on monocytes, their morphology differs from the unique shape of this population. The close contact with nerve bundles, alignment along these fibers, and elongated morphology suggest that these cells are likely sympathetic NAMs (Pirzgalska et al. 2017) (Figure 5B,D and F). To further investigate the functionality of these NAMs, which, to the best of our knowledge, have not been specifically described in the splenic nerve, we induced an inflammatory stimulus by administering lipopolysaccharide (LPS) intraperitoneally. We performed a quantitative analysis to assess their density in the splenic nerve (Figure 5G) and the mean total area of NAMs (Figure 5H). Additionally, we measured the area of CD68 within the NAMs (Figure 5I), as CD68 is a lysosomal membrane marker often associated with activation and phagocytosis. While we observed no significant effect of LPS on the numbers or size of the NAMs (Figure 5G,H), we did observe an increased area of CD68 expression within NAMs (Figure 5I).

Next, we isolated the spleen and performed immunofluorescence and tissue clearing to visualize the splenic nerve insertions into the spleen and the overall distribution of nerve fibers and terminals throughout the spleen parenchyma (Figure 5C). The distribution of neuronal terminals in the spleen appears to be associated with the localization of the blood vessels and capillaries (Figure 5C). Their anatomic appearance varies depending on the distribution of the nerve fibers and the orientation of the vessels. In conventional immunofluorescence of sectioned tissue, the nerve fibers can appear longitudinal if the sectioning is along the blood vessel or as a punctate‐like staining around the vessel if the visualization is of a cross‐sectioned vessel displaying its lumen.

### 3.3 Tracing of Spleen‐Projecting Neurons

**FIGURE 6:**
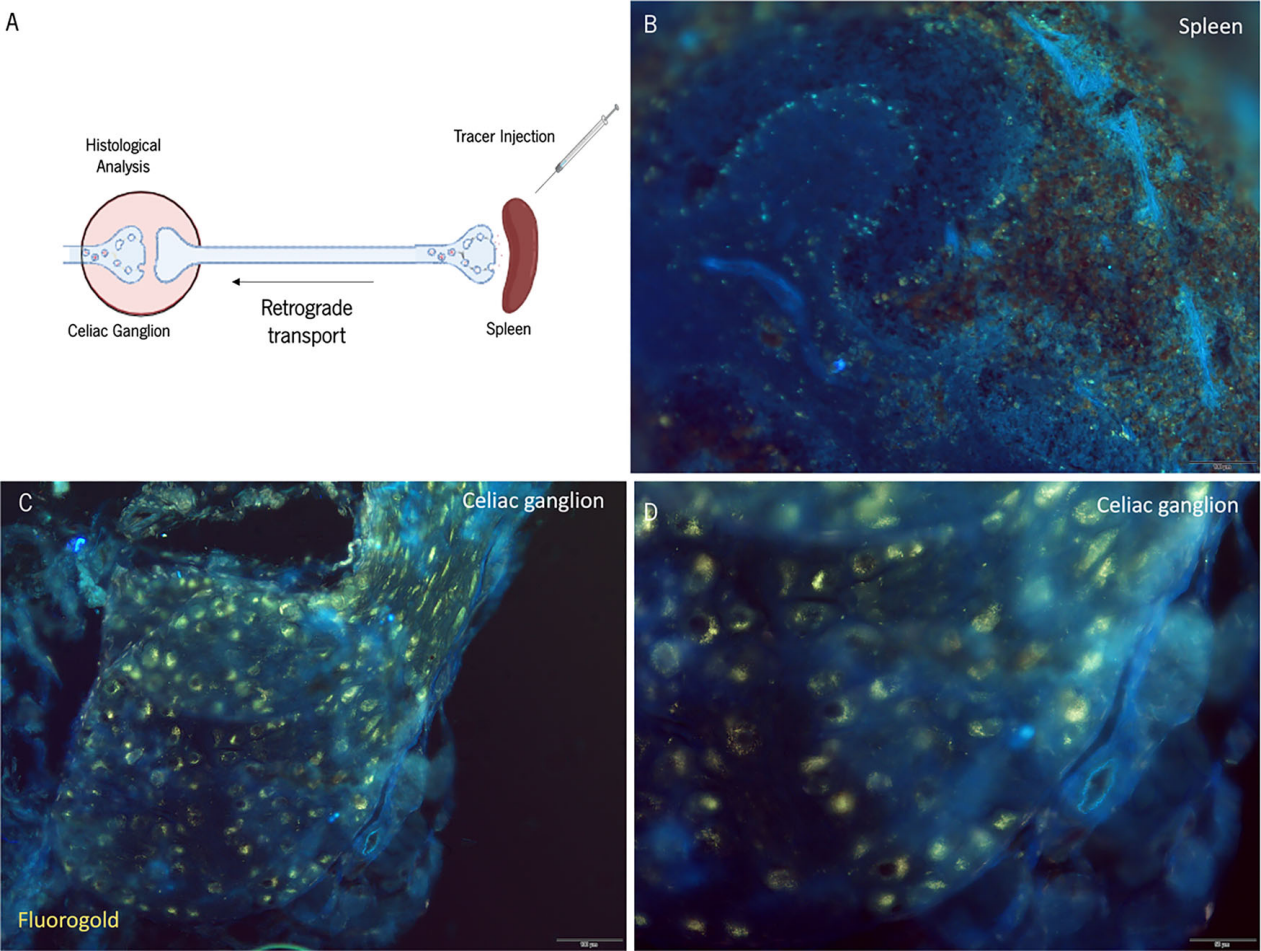
Tracing of spleen‐projecting postganglionic neurons with Fluorogold. (A) Schematic representation of the experimental layout. Fluorogold was injected into the spleen, where postganglionic neuron terminals are located. Created with BioRender. (B) 1 week after the Fluorogold injection, only residual labeling was found at the spleen. Tissue autofluorescence can be observed in cyan blue (C and D). Cell bodies at the celiac ganglion were labelled with fluorogold.

When trying to identify the celiac ganglion precisely, relying solely on its microstructure is insufficient, considering the microscopic structural similarities observed across various ganglia. To unequivocally confirm the identity of the celiac ganglion, we traced the connectivity of its projections to the spleen by using neuronal tracers. The initial approach consisted of injecting a retrograde tracer (Fluorogold) into the spleen. Following uptake by the nerve terminals, the tracer is expected to be transported to the cell soma (Figure 6A).

We isolated and screened the celiac ganglion for Fluorogold staining to confirm its connectivity to the spleen. Gold‐like staining within large cell somas confirmed the spleen connectivity (Figure 6C,D). Only a residual staining was detected in spleen sections, most likely due to the complete transport of the tracer to the celiac ganglion (Figure 6B). To ensure the specificity of the retrograde tracing and rule out nonspecific labeling (i.e., due to leakage and systemic transport of the tracer through the blood), we examined other organs, structures, and tissues, including the peritoneum, mesentery, stellate ganglion, liver, kidney, and heart (Figure S1). No staining was observed in these controls (Figure S1), confirming that the retrograde tracing was specific to the celiac ganglion.

**FIGURE 7:**
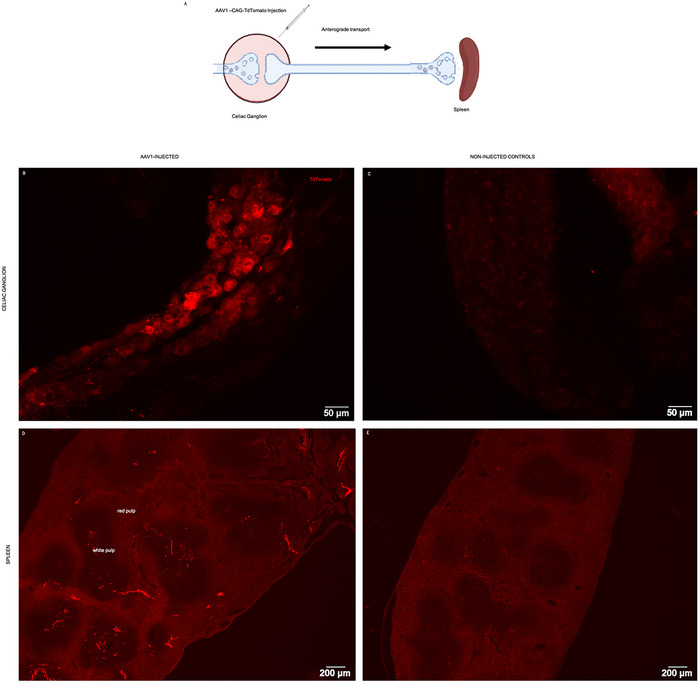
Tracing of spleen‐projecting postganglionic neurons with AAV1‐CAG‐TdTomato. (A) Schematic representation of the experimental layout. AAV1 was injected into the celiac ganglion. After 2 months, the celiac ganglion, the spleen, and the stellate ganglion were collected and screened for TdTomato fluorescent labeling. Created with BioRender. (B) Neuronal cell bodies at the celiac ganglion from AAV1‐injected animals display positive TdTomato labeling. (C) No TdTomato‐positive fluorescence was found in the celiac ganglion from control animals. (D) Intense TdTomato labeling was found on nerve fibers throughout the spleen parenchyma. (E) No TdTomato‐positive fluorescence was found in the spleen from control animals.

A second approach was to inject an anterograde tracer in the celiac ganglion, allowing its visualization in the terminals of spleen‐innervating postganglionic neurons present in the spleen parenchyma (Figure 7A). For that, we used the injection of an AAV with anterograde capability (AAV1‐CAG‐TdTOMATO) into the celiac ganglion (Figure 7A). It is expected that after anterograde transport, transduced cells express the fluorochrome TdTomato at their terminals.

At 2 months post tracer injection, we were able to visualize TdTomato positive labeling in the celiac ganglion of injected mice, on neuronal cell bodies (Figure 7B). Still, not in the control animals (Figure 7C).

In the spleen, we observed a Td‐Tomato‐positive fiber‐like staining (Figure 7D), similar to the TH immunofluorescence. We confirmed that this staining was specific, due to negative labeling in the spleen of a non‐injected animal used as a control (Figure 7E). We excluded nonspecific viral spread (e.g., through the bloodstream) to other structures by analyzing TdTomato labeling in the stellate ganglion—an irrelevant ganglion for the splenic neuronal circuitry. There was no staining in the stellate ganglion (Figure S2).

## 4 Discussion

There is a growing interest in the sympathetic neuronal activity of the spleen, due to its potential to modulate immune responses (Bellinger et al. 2008). However, the precise localization and complex structure of spleen‐innervating neurons represent a challenge for their modulation.

This article describes the dissection procedure to locate the celiac ganglia containing the spleen‐innervating neuron cell soma. Since the celiac ganglia in the mouse can be difficult to observe even under a dissecting microscope, it is advisable to train the access in the rat. This is a larger model, presenting an anatomy similar to that of the mouse, facilitating the identification of the celiac ganglion. In this article, we performed a comparative histological study using celiac ganglia isolated from the mouse and rat and confirmed their anatomical similarity at a microstructural level.

The anatomical approach described here proved particularly helpful for collecting the left celiac ganglion. Accessing the right celiac ganglion may depend on the depth of the dissection/collection, given its lower positioning. Therefore, if the primary objective involves dissecting the right celiac ganglion, an alternative anatomical approach may be advisable for improved access. The left celiac ganglion has improved relevance for the purpose of the present article, since the projecting axons from this ganglion are described to accompany the splenic artery, while the right celiac ganglion accompanies the hepatic artery (Jean Pierre 2017). Indeed, using iDISCO with tissue clearing and PRV retrograde staining, researchers have shown that the hepatic projections come largely from the right celiac ganglion (Torres et al. 2021), in line with the fact that these ganglion projections are irrelevant for the sympathetic innervation of the spleen.

We then followed the celiac ganglion projections by isolating the first segment of the splenic artery. We could observe TH^+^ fibers around this blood vessel, some organized in large thick nerve bundles and other fine fibers. The anatomical approach here described, by exposing the first segment of the splenic artery before its first bifurcation towards the spleen, is particularly useful for the precise ablation of the spleen projections (Carnevale et al. 2016; Monteiro et al. 2020).

Interestingly, we observed CX3CR1^+^ cells associated with these nerve bundles surrounding the splenic nerve. Due to their CX3CR1 expression, macrophage‐like morphology, and their association with the nerve, we hypothesize that these could be sympathetic NAMs that were first identified in adipose tissue sympathetic innervation (Pirzgalska et al. 2017). NAMs were shown to have a central role in NE metabolism, and therefore, may represent an interesting cellular target for modulating the splenic nerve noradrenergic transmission. Although NAMs have been identified in several other nerves, including those innervating adipose tissue, to the best of our knowledge, this is the first time they are described in association with the splenic nerve. In addition, we assessed the functionality of these NAMs by analyzing their response to LPS. NAMs from LPS‐injected animals exhibited a higher area of CD68 staining, suggesting enhanced phagocytic activity. This observation supports the hypothesis that these cells play an active role in inflammatory environments. However, further studies should be conducted to confirm their role, specifically if they are also involved in NE metabolism. It is also important to note that our experiments were conducted exclusively in male mice. While this allowed for consistency within a limited exploratory design, future studies should address potential sex differences in NAM function, particularly given the known influence of sex on inflammatory responses.

In this article, we also employed neuronal tracing techniques to demonstrate the connectivity of the celiac ganglion with the spleen by using an anterograde and a retrograde approach. These experiments were important to confirm the correct identity of the isolated ganglion, given the proximity and physical connectivity of the celiac ganglia with the superior mesenteric and renal ganglia (collectively termed aorticorenal ganglia) and similar microstructure appearance. Our pattern of retrograde labeling in the celiac ganglion is consistent with prior reports in mice (Kressel et al. 2020). In contrast, Bratton and colleagues (Bratton et al. 2012), using rats, reported fewer splenic‐projecting neurons in the celiac ganglion and more in the suprarenal ganglia. These differences likely reflect species‐specific variations in splenic innervation.

Furthermore, we also demonstrated that with this anatomical approach, it is possible to successfully “freehandedly” inject tracers or viral vectors at the left celiac ganglion or in the spleen. Our results confirm the previously reported ability of celiac ganglia neurons to transduce AAV1 (Hammond and Kreulen 2016), in addition to AAV6 (Hammond and Kreulen 2016) and the retrograde AAV2 (T. Zhang et al. 2022). This anatomical approach may then be used for the integration of viral vectors, enabling sophisticated research techniques like chemogenetics or optogenetics, aiming at modulating splenic nerve activity. Another interesting future possibility is combining the retrograde tracing approach described here with genome‐wide or sequencing‐based analyses that could unravel a specific molecular/chemical signature of spleen‐specific celiac neurons.

Of note, the splenic nerve periarterial plexus varies among species (Donegà et al. 2021). In mice and rats, the splenic artery, after stemming from the celiac artery, quickly divides into two main branches. In pigs and humans, the splenic artery courses as a single vessel for a longer distance until it reaches the hilum, where it branches (Donegà et al. 2021). When it comes to the sympathetic innervation of the spleen, humans present a more restricted pattern of innervation of the spleen in comparison to rodents (Verlinden et al. 2019; Cleypool et al. 2021). Between humans and porcine, noradrenergic innervation is abundant in all regions of porcine spleen, while in humans it is mostly restricted to the superior perivascular region (Kirkland et al. 2022). Besides anatomical differences, the splenic nerve stimulation thresholds vary across species (Donegà et al. 2021), emphasizing the importance of considering these distinctions when translating findings from rodent models to humans.

Collectively, the findings presented in this article will offer crucial support for researchers from different fields with a common interest in investigating the role of spleen‐innervating neurons using advanced cellular, molecular, and genetic techniques. This includes applications such as in vitro celiac ganglia culture, isolation of ganglia for sequencing, viral tracing, and the utilization of neuromodulatory techniques such as chemogenetic or optogenetic control, which may contribute to a better and more detailed knowledge of the sympathetic regulation of the spleen, inflammation, and immune function.

## Author Contributions

**Maria Moura**: investigation, writing – original draft, visualization. **Alice Miranda**: methodology, investigation, writing – review and editing. **Jonas Campos**: investigation, writing – review and editing. **Andreia G. Pinho**: investigation, writing – review and editing. **Sara Rito‐Fernandes**: investigation. **Carina Soares‐Cunha**: resources. **António J. Salgado**: resources, writing – review and editing. **Nuno A. Silva**: resources, writing – review and editing. **Susana Monteiro**: conceptualization, funding acquisition, writing – original draft, investigation.

## Conflicts of Interest

The authors declare no conflicts of interest.

## Peer Review

The peer review history for this article is available at https://publons.com/publon/10.1002/cne.70086

## Supporting information



Figure S1—The celiac ganglion, spleen, peritoneum, heart, mesentery, stellate ganglion, liver, and kidney were collected to control for nonspecific Fluorogold labeling. Strong labeling was localized to cell bodies in the celiac ganglion (A), and some labeling was found at the site of injection in the spleen. (B) No staining was found in any other organ or tissue collected and used as controls (C–H).
Figure S2—The stellate ganglion was collected as an irrelevant ganglion for the spleen‐projecting neurons to control for nonspecific systemic AAV delivery. No staining was found in the stellate ganglion of AAV‐1‐injected animals.

## Data Availability

Data are available upon reasonable request from the authors.
