# Supplementary figures and images for: Macro‐ and Microanatomy of the Sympathetic Innervation of the Spleen in Rodents
Source: J Comp Neurol. 2025 Aug 29;533(9):e70086. doi: 10.1002/cne.70086 (PMC12396320; doi:10.1002/cne.70086)

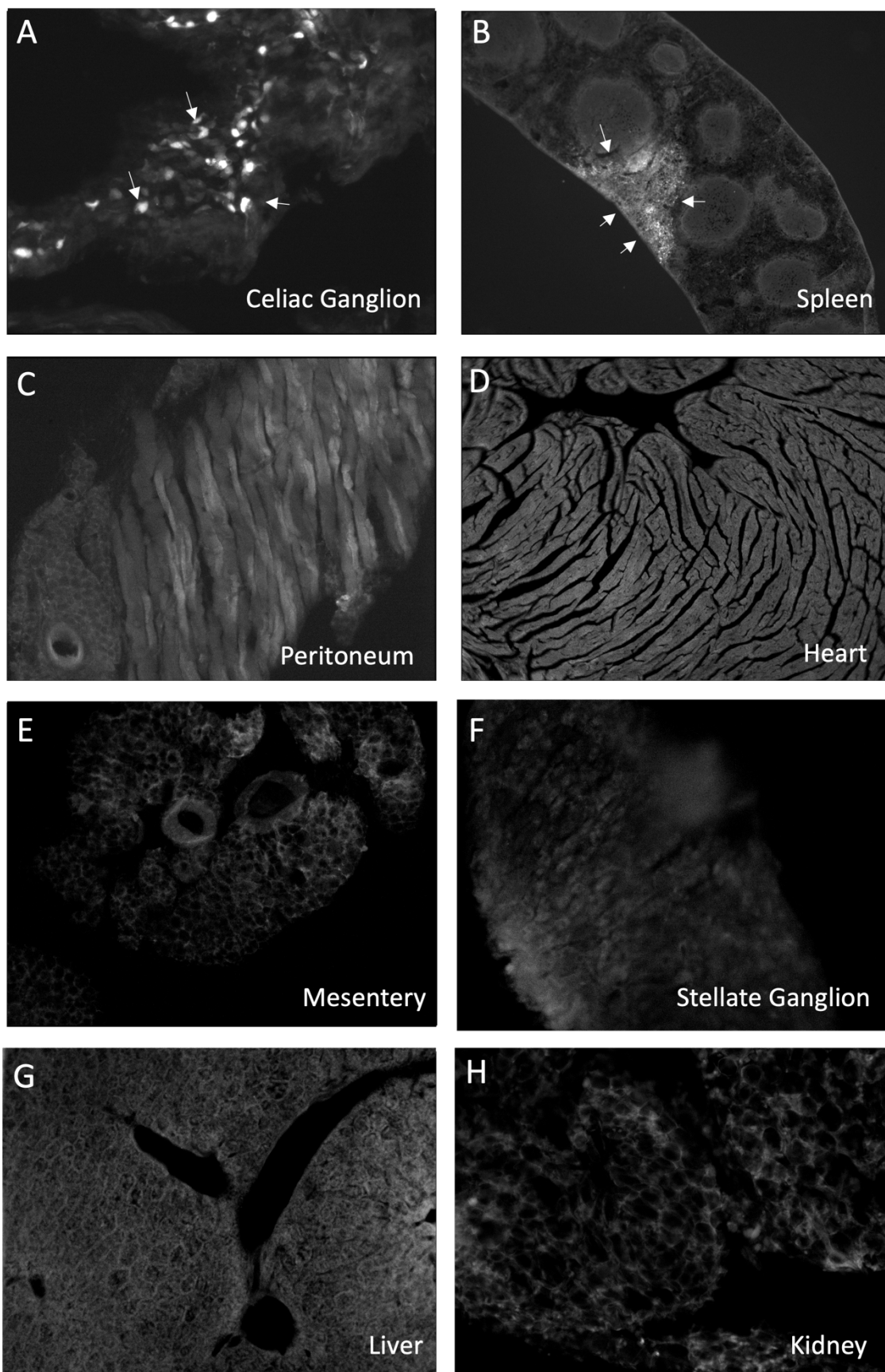

Fig S1

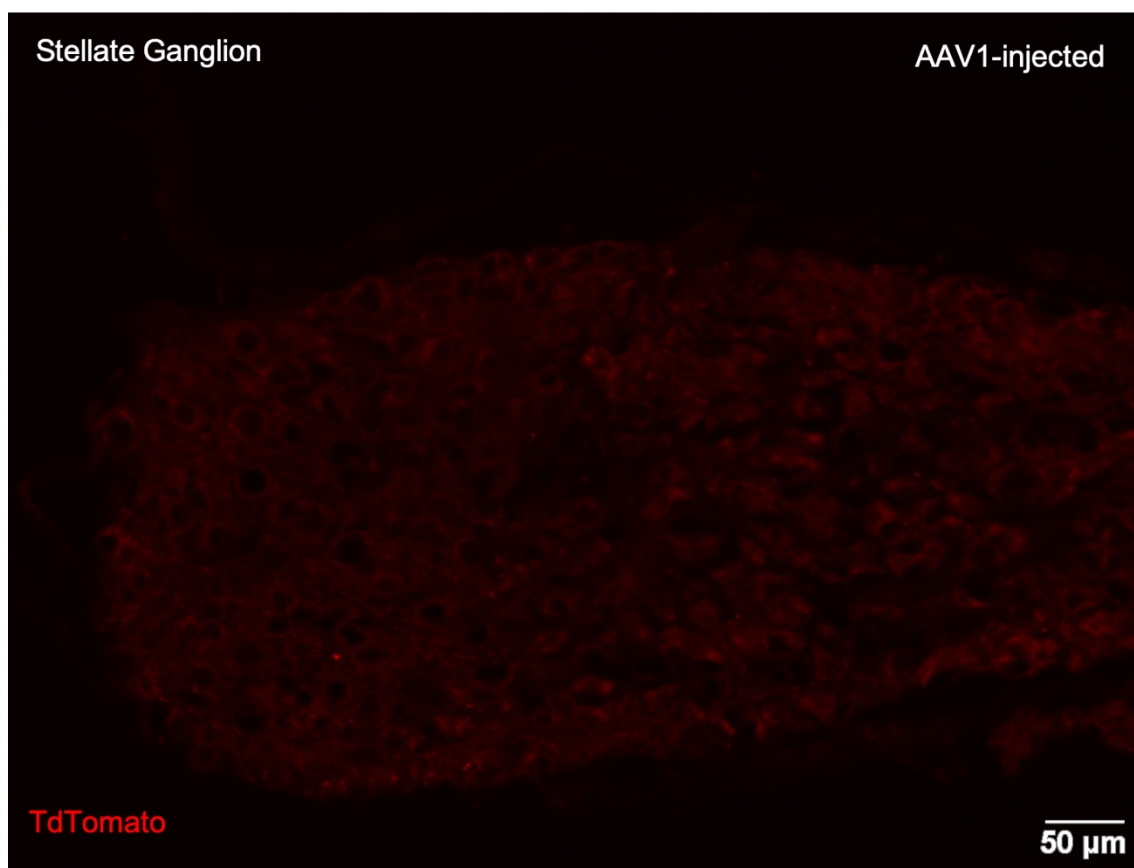

Fig. S2

Supplement: Supplementary file 1 — Figure S1—The celiac ganglion, spleen, peritoneum, heart, mesentery, stellate ganglion, liver, and kidney were collected to control for nonspecific Fluorogold labeling. Strong labeling was localized to cell bodies in the celiac ganglion (A), and some labeling was found at the site of injection in the spleen. (B) No staining was found in any other organ or tissue collected and used as controls (C–H). Figure S2—The stellate ganglion was collected as an irrelevant ganglion for the spleen‐projecting neurons to control for nonspecific systemic AAV delivery. No staining was found in the stellate ganglion of AAV‐1‐injected animals. [file CNE-533-e70086-s001.pdf]
